# MicroRNA-4429 suppresses proliferation of prostate cancer cells by targeting distal-less homeobox 1 and inactivating the Wnt/β-catenin pathway

**DOI:** 10.1186/s12894-021-00810-x

**Published:** 2021-03-19

**Authors:** Jinguo Wang, Sheng Xie, Jun Liu, Tao Li, Wanrong Wang, Ziping Xie

**Affiliations:** Department of Andrology, Renmin Hospital, Hubei University of Medicine, No. 39 Chaoyang Middle Road, Maojian District, Shiyan, 442000 Hubei People’s Republic of China

**Keywords:** Prostate cancer, MicroRNA-4429, Wnt/β-catenin pathway, DLX1, Biological behavior

## Abstract

**Background:**

Emerging evidence suggests that microRNAs (miRNAs) play multiple roles in human cancers through regulating mRNAs and distinct pathways. This paper focused on the functions of miR-4429 in prostate cancer (PCa) progression and the molecules involved.

**Methods:**

Expression of miR-4429 in PCa tissues and cells was determined. Upregulation of miR-4429 was introduced in PCa cells to examine its role in the malignant behaviors of cells. The putative target mRNA of miR-4429 involved in PCa progression was predicted from a bioinformatic system and validated through luciferase assays. Overexpression of distal-less homeobox 1 (DLX1) was further induced in cells to validate its implication in miR-4429-mediated events. The activity of Wnt/β-catenin pathway was determined.

**Results:**

miR-4429 was poorly expressed in PCa tissues and cells. Artificial upregulation of miR-4429 significantly reduced proliferation, growth, invasion, migration and resistance to death of cancer cells and inactivated the Wnt/β-catenin pathway. DLX1 mRNA was found as a target of miR-4429. Upregulation of DLX1 restored the malignant behaviors of PCa cells which were initially suppressed by miR-4429, and it activated the Wnt/β-catenin pathway.

**Conclusion:**

Our study highlights that miR-4429 inhibits the growth of PCa cells by down-regulating DLX1 and inactivating the Wnt/β-catenin pathway. This finding may offer novel insights into PCa treatment.

**Supplementary Information:**

The online version contains supplementary material available at 10.1186/s12894-021-00810-x.

## Background

Prostate cancer (PCa) is the second most common malignant tumor and the sixth leading cause of cancer-associated death among males around the world [1]. Recently, the incidence of PCa has been elevating steadily in China with the economy improvement [2]. Established risk factors for PCa comprise black race, advancing age, family history and certain genetic polymorphisms [3, 4]. Currently, digital rectal examination, prostate-specific antigen testing and prostate needle biopsies are applied in the diagnosis and monitoring of PCa progression [5]. It is essential to identify more biomarkers for PCa prediction and treatment, which requires a better recognition of the molecular pathogenesis of the disease.

MicroRNAs (miRNA) are a major type of small noncoding RNAs that can negatively modulate gene expression via binding to the 3ʹuntranslated region (3ʹUTR) of target mRNAs [6]. The miRNAs play versatile roles in many key cellular processes, such as carcinogenesis, proliferation and metastasis [7]. Aberrant expression of miRNAs, either upregulation or downregulation, has been frequently found in PCa tissues versus normal tissues [5]. miR-4429 was discovered to have a suppressive effect on several types of malignancies such as thyroid cancer [8] and cervical cancer [9]. However, there is little evidence concerning its role in the tumorigenesis of PCa. Distal-less homeobox 1 (DLX1) was firstly found in Drosophila melanogaster and found to mediate development of nerves and embryo [10]. DLX1 downregulation has been demonstrated to lead to PCa cell growth arrest, revealing that DLX1 might serve as an oncogene [11]. Wnt signaling is a pivotal pathway controlling adult tissue homeostasis and β-catenin is an essential effector of this signaling [12]. The Wnt/β-catenin pathway exerts key functions in a variety of cellular processes such as cell fate determination, cell growth and polarity, and stem cell maintenance [13]. This pathway has been reported to induce invasive behaviors of PCa cells [14]. In this study, DLX1 was confirmed as a target of miR-4429. We hypothesized that miR-4429 could inhibit PCa progression through regulating DLX1 and the Wnt/β-catenin pathway, with gain- and loss-of function studies of these molecules performed to validate this hypothesis.

## Methods

### Ethical approval

This study was ratified by the Ethics Committee of Renmin Hospital, Hubei University of Medicine and performed in line with the *Declaration of Helsinki*. Signed informed consent form was obtained from each eligible participant.

### Collection of clinical samples

Biopsy specimens of primary PCa tissues and the adjacent normal prostate tissues were collected from 35 patients with PCa in People’s Hospital Affiliated to Hubei University of Medicine from February 2017 to November 2019. The patients aged from 42 to 68 years at a median age of 57 years. The tissues were stored at -80 °C until further use. The inclusion criteria were: 1. Patients were diagnosed with PCa by prostate puncture, digital rectal examination, computerized tomography or magnetic resonance imaging, or prostate specific antigen test; 2. Patients aged between 40 and 70 years; 3. Gleason score was not less than 6. Patients with a history of hormone therapy, radiotherapy, chemotherapy or surgery, or those with other malignancies were excluded.

### Cell culture

PCa cell lines PC-3, LNCAP, and DU145 and a human normal prostate epithelial cell line RWPE-1 were procured from the American Standard Bacterial Bank (ATCC, Rockville, Maryland, USA). Cells were cultured in RPMI-1640 medium (Gibco, Gaithersburg, MD, USA) supplemented with 10% fetal bovine serum (FBS) in a 37℃ incubator with 5% CO_2_.

### Reverse transcription quantitative polymerase chain reaction (RT-qPCR)

Total RNA in cells and tissues was extracted using a TRIzol kit (TAKARA, Shiga, Japan). A SYBR Green qPCR Mix Kit (Thermo Fisher Scientific, Waltham, MA, USA) was used for quantitative PCR after the addition of templates and primers. The gene expression was detected on the ABI real-time PCR platform (Thermo Fisher Scientific). Primers were purchased from Invitrogen (Shanghai, China). Internal references were U6 (for miRNA) and glyceraldehyde phosphate dehydrogenase (GAPDH) (for mRNA). The primer sequences are as follows: miR-4429 (GenBank ACCESSION: NR_039627.1): forward primer: 5ʹ-GGCCAGGCAGTCTGAGTTG-3ʹ; reverse primer: 5ʹ-GGGAGAAAAGCTGGGCTGAG-3ʹ. DLX1 (GenBank ACCESSION: NM_178120): forward primer: 5ʹ-GCGGCCTCTTTGGGACTCACA-3ʹ; reverse primer: 5ʹ-GGCCAACGCACTACCCTCCAGA-3ʹ. GAPDH (GenBank ACCESSION: NM_001289746.2): forward primer: 5ʹ-GAGTCCACTGGCGTCTTCAC-3ʹ; reverse primer: 5ʹ-ATCTTGAGGCTGTTGTCATACTTCT-3ʹ. U6 (GenBank ACCESSION: NR_004394.1): forward primer: 5ʹ-CTCGCTTCGGCAGCACA-3ʹ; reverse primer: 5ʹ-AACGCTTCACGAATTTGCGT-3ʹ. Axin2 (GenBank ACCESSION: NM_004655.4): forward primer: 5ʹ-CAGATCCGAGAGGATGAAGAGA-3ʹ; reverse primer: 5ʹ-AGTATCGT CTGCGGGTCTTC-3ʹ. CD44 (GenBank ACCESSION: NM_000610.4): forward primer: 5ʹ-ACCCTCCCCTCATTCACCAT-3ʹ; reverse primer: 5ʹ-GTTGTACTACTAGGAGTTGCCTGGATT-3ʹ. Relative gene expression was examined by the 2^−∆∆Ct^ method.

### Cell apoptosis detection

Exponentially growing cells were seeded in 6-well plates, and the cells were rinsed twice with pre-chilled phosphate-buffered saline (PBS) and stained using a PI/Annexin-V kit at room temperature without light exposure for 15 min. Then, the cells were appended with 300 μL 1 × binding buffer and suspended, and transferred into a 5-mL flow tube. A flow cytometer (Thermo Fisher Scientific) was adopted to detect the number of apoptotic cells within one hour.

### Cell transfection

DU145 and PC-3 cells in good growth conditions were sorted in 6-well plates (4 × 10^5^ cells/mL) and incubated in DMEM with 10% FBS for 24 h of pre-transfection. Next, the cells were introduced with miR-4429 mimic/inhibitor and the corresponding miR-4429 mimic/inhibitor control, pcDNA-DLX1, or empty pcDNA (all procured from Thermo Fisher Scientific). Transfection was conducted using Lipofectamine 2000 reagent (Thermo Fisher Scientific) according to the manufacturer’s protocols. Forty-eight hours later, cells were harvested for subsequent experiments, and miR-4429 expression was detected by RT-qPCR to evaluate the transfection efficiency.

### Determination of cell proliferation

A cell counting kit-8 (CCK-8) assay was performed to evaluate cell proliferation. In brief, cells were treated with 10 μL/well CCK-8 solution (Beyotime Biotechnology, Shanghai, China) and cultured at 37 °C for 2 h. The optical density at 450 nm was measured with a microplate reader (BioTek Instruments).

### Colony formation assay

Cells were detached in trypsin, seeded onto 6-well plates (200 cells/well), and then incubated in DMEM with 10% FBS for 2 w. The medium was renewed every three days. Next, cells were fixed in methanol for 15 min and then stained with crystal violet (1%) for 30 min. The images (containing more than 50 cells/colonies) were captured using a light microscope (magnification: 100 × ; Olympus Optical Co., Ltd, Tokyo, Japan) and the number of cell colonies was counted.

### Cell invasion assay

The Transwell apical chamber was pre-coated with Matrigel (BD Biosciences, Franklin Lakes, NJ, USA) under sterile conditions. Then, each apical chamber was loaded with 30 μL of serum-free RPMI 1640 medium, and then placed in a CO_2_ incubator for later use. Each basolateral chamber was loaded with 200 μL cell suspension and 500 μL RPMI 1640 medium containing 10% FBS. The chambers were incubated in a 37℃ incubator with 5% CO_2_ for 24 h. After that, the non-invaded cells were wiped with a cotton swab, and cells invaded to the lower membranes were fixed in 4% paraformaldehyde and stained by crystal violet for 10 min. The count of invading cells was evaluated under a light microscope.

### Luciferase activity assay

The binding sequence between miR-4429 and DLX1 mRNA was first predicted on a bioinformatic system Starbase (http://starbase.sysu.edu.cn/). To validate the binding relationship between miR-4429 and DLX1 mRNA, DLX1 3′UTR containing the putative binding site with miR-4429 was designed and inserted into pmirGLO reporter vectors (LMAIBio Co., Ltd., Shanghai, China) to construct wild-type luciferase vectors. A mutant type vector was designed by mutating the binding sequence between miR-4429 and DLX1 mRNA. Well-constructed reporter vectors were co-transfected with miR-4429 mimic or mimic control into PCa cells. Forty-eight hours later, the cells were harvested and lysed, and luciferase activity in cells was determined on a dual luciferase reporting system (Promega Corp., Madison, Wisconsin, USA) according to the manufacturer's protocol.

### Western blot analysis

Cells were lysed in Western and IP cell lysis buffer (Beyotime) to extract total protein, and the protein concentration was determined using a bicinchoninic acid kit (No. P0009, Beyotime). Next, the proteins were separated by polyacrylamide gel (5% spacer gel and 12% separation gel), transferred on polyvinylidene fluoride membranes and treated with 5% bovine serum albumin to block the non-specific binding. After that, the membranes were co-incubated with the primary antibodies at 4℃ overnight and then with the secondary antibody at 37℃ for 2 h. Next, the protein bands were developed using enhanced chemiluminescence reagent (WBKLS0100, Millipore, Billerica, MA, USA). Relative gray value analysis was performed on all immunoblotted bands, and the ratio of the gray value of the target band to the internal reference band was considered as the relative expression of the protein. Primary antibodies included DLX1 (UniProt Entry ID: P56177) (1:2000, ab126054), β-catenin (UniProt Entry ID: P35222) (1: 7500, ab32572) and GAPDH (1: 2500, ab9485) and secondary antibody was (1:50,000, ab205718) (Abcam, NY, USA).

### Statistical analysis

Data were processed using the SPSS 21.0 (IBM Corp. Armonk, NY, USA) statistical software. Results were shown as mean ± standard deviation (SD) from three independent experiments. The two-group comparison was analyzed by the *t* test. Comparisons among three or more groups were implemented using one-way analysis of variance (ANOVA) or two-way ANOVA. Pairwise comparison after ANOVA was implemented using Tukey's multiple comparisons test. *P* less than 0.05 was considered statistically significant.

## Results

### miR-4429 is poorly expressed in PCa cells and tissues

**Fig. 1:**
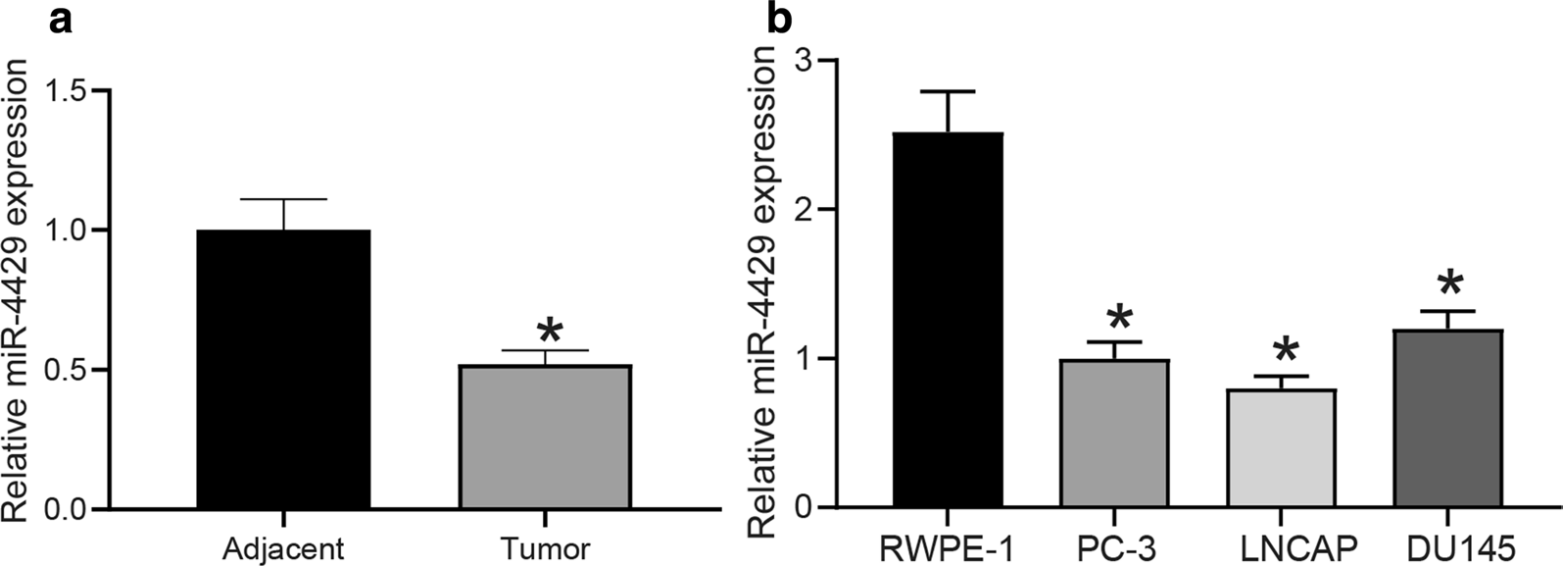
miR-4429 is lowly expressed in PCa cells and tissues. **a** miR-4429 expression in 35 pairs of PCa tissues and the adjacent normal tissues determined by RT-qPCR (paired *t* test, *P* = 0.0023). **b** miR-4429 expression in PCa cell lines (PC-3, LNCAP and DU145) and human normal prostate epithelial cell line RWPE-1 determined by RT-qPCR (one-way ANOVA, *P* < 0.0001). Data were expressed as mean ± SD from three independent experiments. *P < 0.05 was considered to show statistical significance

First, the miR-4429 expression in PCa tissues and cells was detected. Compared to the adjacent normal tissues, the expression of miR-4429 was significantly reduced in PCa tissues (Fig. 1a). Compared to that in human normal prostate epithelial cell line RWPE-1, the expression of miR-4429 was also declined in the PCa cell lines (PC-3, LNCAP and DU145) (Fig. 1b).

### miR-4429 mimic suppresses the biological activity of PCa cells

**Fig. 2:**
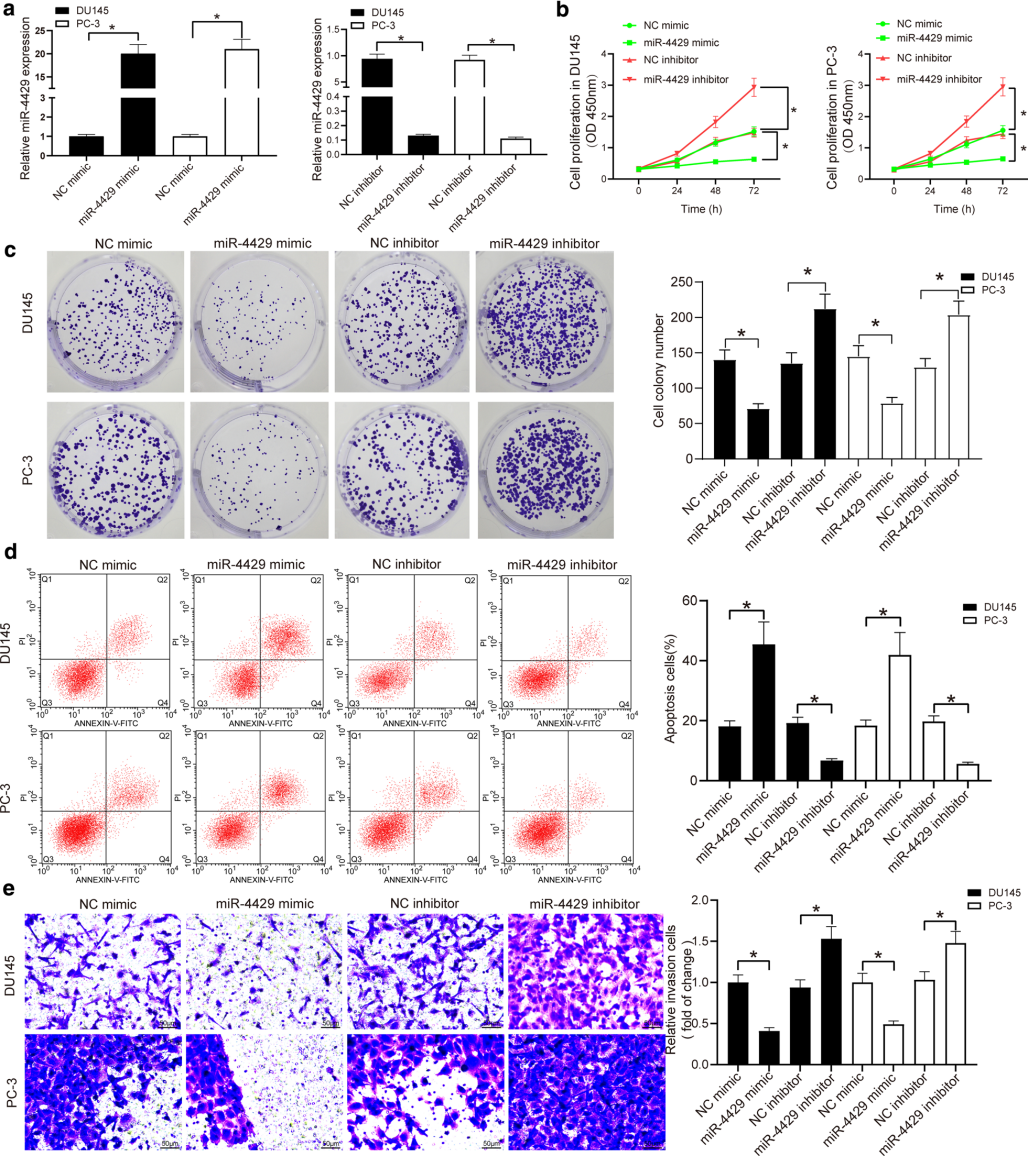
miR-4429 mimic suppresses the biological activity of PCa cells. **a** miR-4429 expression in DU145 and PC-3 cells introduced with miR-4429 mimic/miR-4429 inhibitor determined by RT-qPCR (unpaired *t* test, *P* < 0.0001). **b** PCa cell proliferation in DU145 and PC-3 cells introduced with miR-4429 mimic/miR-4429 inhibitor determined by CCK-8 assay (two-way ANOVA, *P* < 0.0001). **c** Number of colonies in DU145 and PC-3 cells introduced with miR-4429 mimic/miR-4429 inhibitor determined by colony formation assay (one-way ANOVA, *P* < 0.0001). **d** Apoptosis in DU145 and PC-3 cells introduced with miR-4429 mimic/miR-4429 inhibitor determined by flow cytometry (one-way ANOVA, *P* < 0.0001). **e** Invasion ability in DU145 and PC-3 cells introduced with miR-4429 mimic/miR-4429 inhibitor determined by Transwell assay (one-way ANOVA, *P* < 0.0001). Data were expressed as mean ± SD from three independent experiments. *P < 0.05 was considered to show statistical significance

Next, to explore the function of miR-4429 in the activity of PCa cells, miR-4429 mimic, miR-4429 inhibitor and the corresponding negative controls (NCs) were administrated into DU145 and PC-3 cells. RT-qPCR analysis showed that miR-4429 expression in cells was significantly elevated by miR-4429 mimic but reduced by miR-4429 inhibitor (Fig. 2a). Then, the CCK-8 and colony formation assays suggested that miR-4429 mimic restricted proliferation and colony formation ability of the PCa cells, while miR-4429 inhibitor led to inverse results (Fig. 2b, c). As for apoptosis and invasion, it was found that the invasion and migration abilities of cells were reduced by miR-4429 mimic but enhanced by miR-4429 inhibitor (Fig. 2d, e). It can be concluded that miR-4429 mimic can repress the biological activity of PCa cells.

### DLX1 is a target mRNA of miR‐4429

**Fig. 3:**
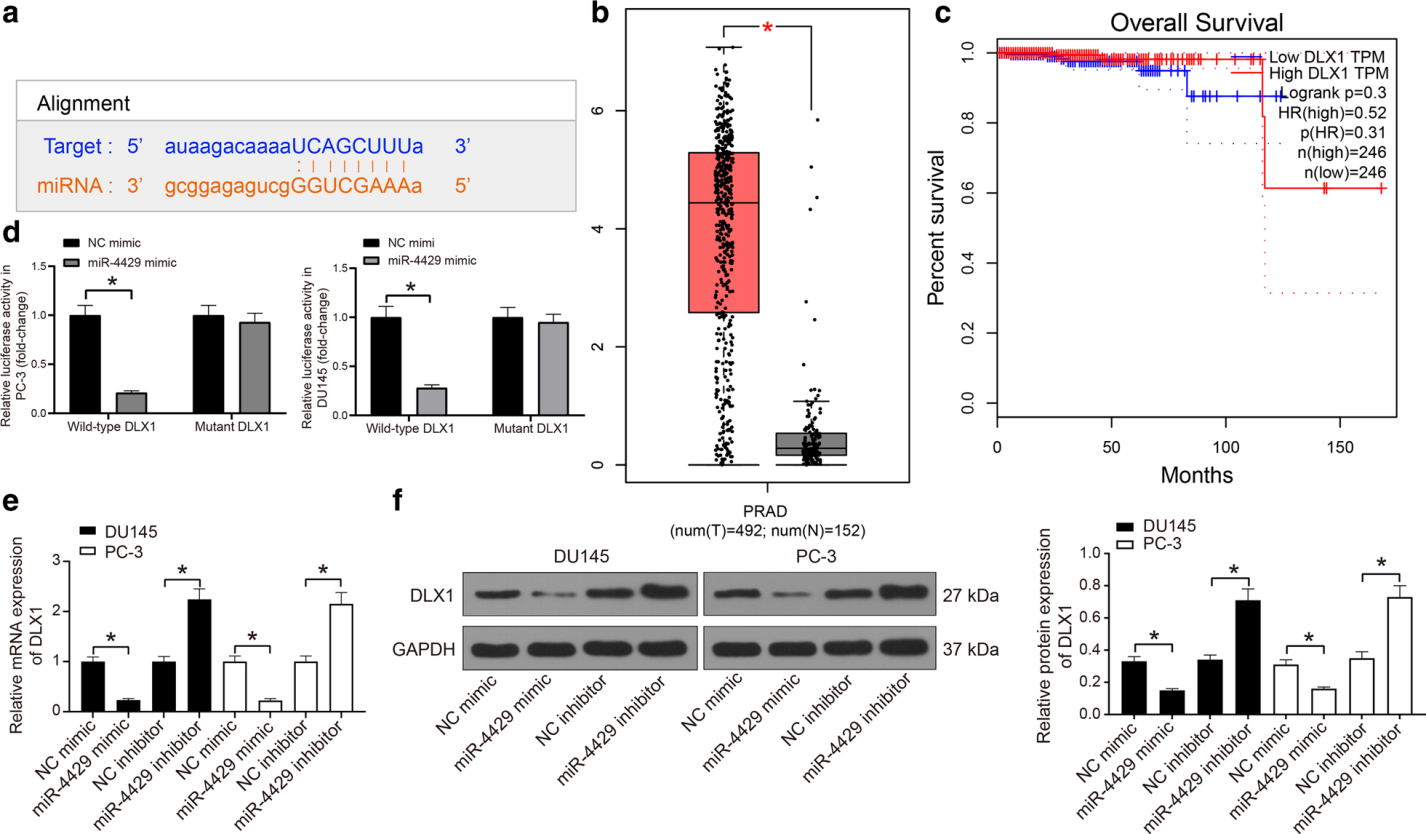
DLX1 is a target gene of miR-4429 in PCa cells. **a** Putative binding site between miR-4429 and the DLX1 3′-UTR. **b**–**c** PCa expression (**b**) and its relevance to the overall survival of patients (**c**) predicted according to the data on the GEPIA database (**d**). Binding relationship between miR-4429 and DLX1 3′-UTR evaluated via a dual luciferase reporter gene analysis. DLX1 3′-UTR luciferase vector and miR-4429 mimic or NC mimic were co-transfected into DU145 and PC-3 cells and incubated for 48 h before measuring luciferase activity (one-way ANOVA, *P* < 0.0001). **e** miR-4429 mimic/inhibitor or NC mimic/inhibitor transfected into DU145 and PC-3 cells for 48 h, and DLX1 mRNA expression was measured by RT-qPCR (one-way ANOVA, *P* < 0.0001). **f** miR-4429 mimic/inhibitor or NC mimic/inhibitor transfected into DU145 and PC-3 cells for 48 h, and DLX1 protein expression was measured by western blot analysis (one-way ANOVA, *P* < 0. 0001) (see original western blots in Additional file 1: Figure S1). Data were expressed as mean ± SD from three independent experiments. *P < 0.05 was considered to show statistical significance

A luciferase assay was implemented to validate the targeting relationship between miR-4429 and DLX1. According to the data from the bioinformatics system StarBase, DLX1 mRNA was suggested as a potential target of tumor-associated miR-4429. DLX1 3′-UTR contains a putative binding site with miR-4429 (Fig. 3a). We then explored the expression profiles of DLX1 in PCa according to the data available from the Gene Expression Profiling Interactive Analysis (GEPIA) dataset (http://gepia.cancer-pku.cn/detail.php?clicktag=degenes###). It was suggested that DLX1 was highly expressed in PCa samples, while its expression showed no significant relevance to the overall survival rate of patients (Fig. 3b). This might be attributed to the individual differences between patients (Fig. 3c).

The binding relationship between miR-4429 and DLX1 mRNA was validated through a luciferase assay. It was found that the luciferase activity of the wild-type DLX1 3′-UTR luciferase vector was significantly reduced by miR-4429 mimic, while the activity of mutant-type luciferase vector was not significantly changed (Fig. 3d). In addition, the RT-qPCR and western blot assay results suggested that the expression of DLX1 in PCa cells was significantly reduced by miR-4429 mimic but elevated by miR-4429 inhibitor (Fig. 3e, f). Taken together, these results indicate that DLX1 is a target mRNA of miR-4429 in PCa cells.

### miR-4429 mimic inhibits Wnt/β-catenin while DLX1 activates this pathway

**Fig. 4:**
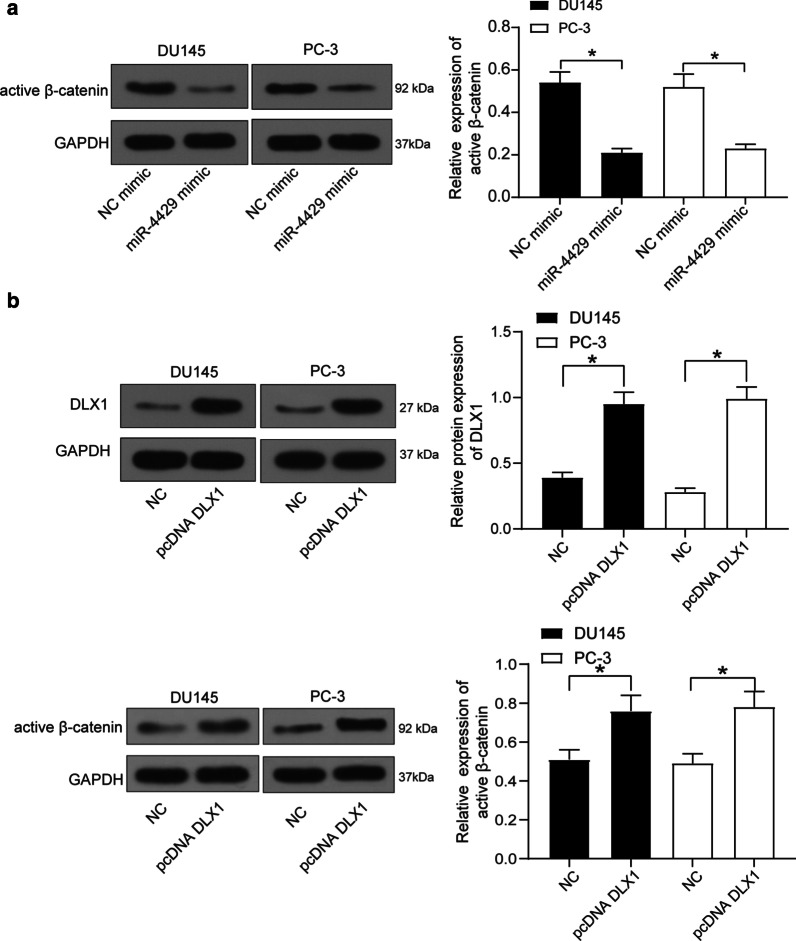
miR-4429 down-regulates the expression of Wnt/β-catenin. **a** protein level of β-catenin after miR-4429 administration determined by western blot analysis (one-way ANOVA, *P* < 0.0001) (see original western blots in Additional file 1: Figure S2); **b** protein level of β-catenin after pcDNA-DLX1 administration determined by western blot analysis (one-way ANOVA, *P* = 0.0009) (see original western blots in Additional file 1: Figure S3). Data were expressed as mean ± SD from three independent experiments. *P < 0.05 was considered to show statistical significance

Since the Wnt/β-catenin pathway is implicated in many human malignancies, we investigated whether the Wnt/β-catenin pathway activation was involved in the events mediated by miR-4429. Importantly, it was found that miR-4429 mimic suppressed the protein level of β-catenin in PCa cells (Fig. 4a). In addition, artificial upregulation of DLX1 was introduced in the PCa cells. In this setting, the protein level of β-catenin in cells was restored (Fig. 4b).

### DLX1 overexpression suppresses the antitumor effect of miR-4429 in PCa cells

**Fig. 5:**
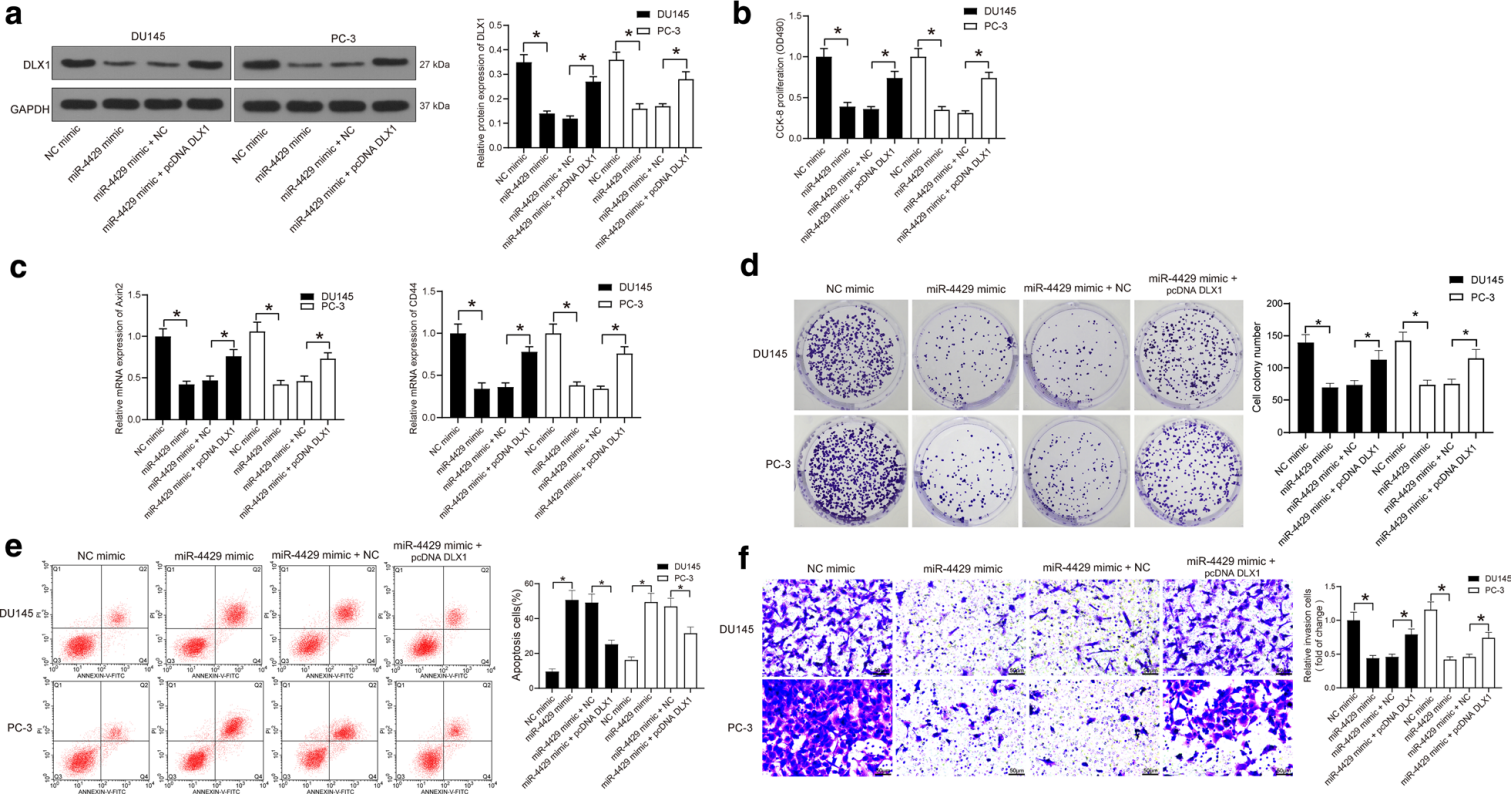
DLX1 blocks the antitumor function of miR-4429 in PCa cells. **a** DLX1 protein level in DU145 and PC-3 cells introduced with miR-4429 mimic and pcDNA DLX1 determined by western blot analysis (one-way ANOVA, *P* < 0.0001) (see original western blots in Additional file 1: Figure S5). **b** Proliferation of DU145 and PC-3 cells introduced with miR-4429 mimic and pcDNA DLX1 determined by CCK-8 assay (one-way ANOVA, *P* < 0.0001). **c** Expression of Axin2 and CD44 in DU145 and PC-3 cells introduced with miR-4429 mimic and PcDNA DLX1 determined by RT-qPCR (one-way ANOVA, *P* < 0.0001). **d** Number of colonies of DU145 and PC-3 cells introduced with miR-4429 mimic and PcDNA DLX1 determined by colony formation assay (one-way ANOVA, *P* < 0.0001). **e** Apoptosis in DU145 and PC-3 cells introduced with miR-4429 mimic and PcDNA DLX1 determined by flow cytometry (one-way ANOVA, *P* < 0.0001). **f** Invasion ability in DU145 and PC-3 cells introduced with miR-4429 mimic and PcDNA DLX1 determined by Transwell assay (one-way ANOVA, *P* < 0.0001). Data were expressed as mean ± SD from three independent experiments. *P < 0.05 was considered to show statistical significance

To explore the involvement of DLX1 inhibition in the miR-4429-mediated events, pcDNA DLX1 was further transfected into DU145 and PC-3 cells after miR-4429 mimic administration. As shown in Fig. 5a, it was found that pcDNA DLX1 partially blocked the inhibitory effect of miR-4429 mimic on DLX1 expression. The proliferation, invasion and colony formation abilities of cells and the expression of PCa-biomarker Axin2 and CD44 in cells initially inhibited by miR-4429 mimic were recovered after further DLX1 overexpression. Accordingly, DLX1 reduced the apoptosis rate in the PCa cells (Fig. 5b–f).

## Discussion

**Fig. 6:**
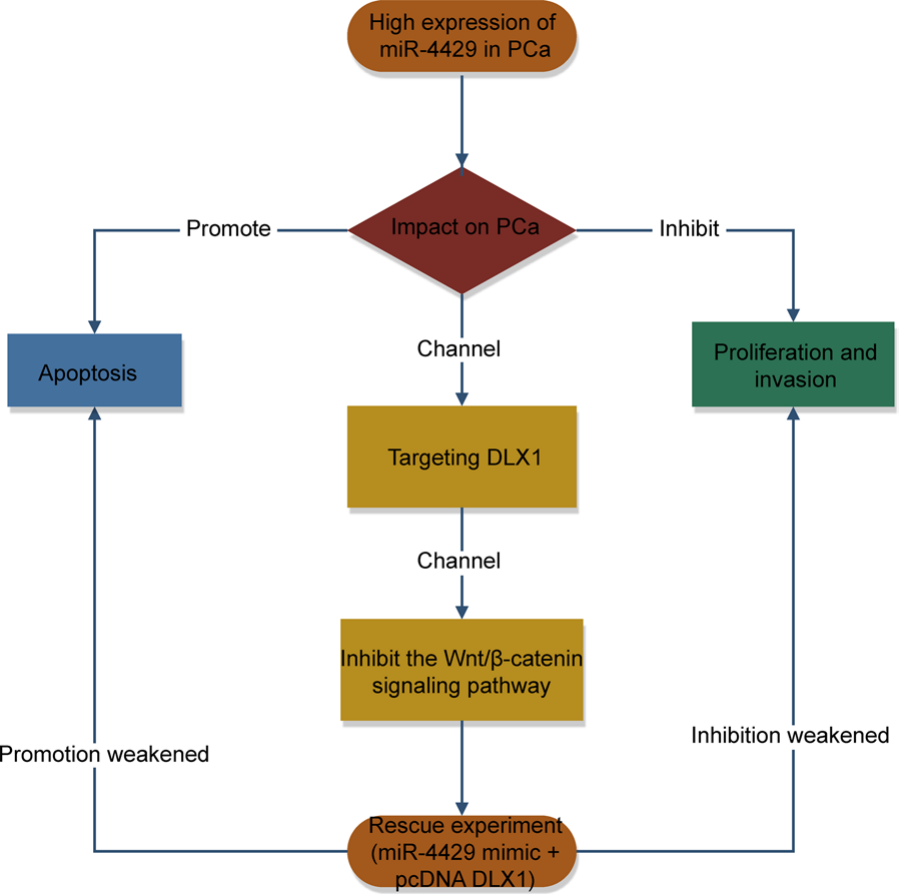
A flow chart for the functions of miR-4429 and DLX1 in PCa cells

In recent years, novel promising PCa-specific targets have been recognized in many researches, while only a few of them have reached clinical practice [15]. It is well-known that both overtreatment and overdiagnosis would be decreased if PCa-specific targets could distinguish inactive from aggressive tumors. Therefore, developing novel biomarkers may help a lot in PCa management. In this article, we confirmed that miR-4429 plays a tumor-suppressive role in PCa via mediating DLX1 and Wnt/β-catenin pathway (Fig. 6).

The miR-4429 has been identified as a tumor suppressor in several kinds of tumors while its role in PCa has not been concerned. Here, the initial finding of this research was that miR-4429 was poorly expressed in both PCa tissue samples and cell lines compared to the normal tissues or cells. Next, artificial up-regulation of miR-4429 significantly reduced proliferation, invasion, colony formation, and resistance to apoptosis of cells. These results were partially in concert with the tumor-suppressing roles of miR-4429 in several previous studies. For instance, miR-4429 was found as a sponge for long non-coding RNA LINC00313 and was expressed at a low lever in thyroid cancer, and its upregulation reduced proliferation and the migratory and colony-forming abilities of thyroid cancer cells [8]. Likewise, miR‐4429 has been reported to be reduced in cervical cancer cells versus normal cell lines and it enhanced the sensitivity of cells to irradiation [9]. Furthermore, miR-4429 has been reported as a tumor suppressor in clear cell renal cell carcinoma (ccRCC), and its downregulation was associated with poor prognosis of ccRCC patients while its upregulation inhibited growth of ccRCC cells [16]. Likewise, silencing of miR-4429 was also found to increase the viability of glioblastoma multiforme cells [17].

Data from the StarBase system suggested that miR-4429 owns a binding relationship with DLX1 mRNA, and the direct binding was confirmed by a luciferase assay. Although the correlation between DLX1 and miR-4922 has never been documented before, the involvements of target mRNAs have been well-established in the antioncogenic role of miR-4922, such as CDK6 downregulation in the above-mentioned ccRCC inhibition [16]. Another study has elucidated that miR-4429 prevented the onset of gastric cancer via targeting METTL3 [18]. As for DLX1, it has been suggested as an important target of FOXM1 to enhance the aggressiveness of ovarian cancer [19]. More relevantly, DLX1 expression has been found to be elevated in PCa tissues, indicating a possible association between DLX1 and PCa progression, [20]. In concert with this, DLX1 has been suggested as a potent biomarker for high‐grade PCa detection [15]. A study by Liang et al*.* suggested that DLX1 was elevated in PCa clinical samples, and DLX1 promoted growth of PCa cells through activating the β-catenin/TCF pathway [21]. Here, our study found that overexpression of DLX1 partially blocked the inhibitory effect of miR-4429 on the PCa cells. In addition, miR-4429 suppressed was found to inactivate the Wnt/β-catenin pathway, while the activity of this pathway was restored after DLX1 upregulation. Aberrant activation of the Wnt/β-catenin is closely associated with onset and progression of multiple human cancers, leaving this signaling as a potential target for cancer therapy [22, 23]. There is no exception for PCa [14, 24]. As reported, distinct miRNAs could directly mediate the Wnt/β-catenin pathway in many malignancies [25, 26]. Wnt signaling aberrant activation is able to induce nuclear β-catenin accumulation, contributing to transcriptional activation of proto-oncogenes that are associated with cell progression [27]. Furthermore, Wnt/β-catenin pathway has been revealed to induce tumor cell malignancy in hormone-refractory PCa cells in a ligand-independent manner [28]. In addition to the finding that miR-4429 inactivated this pathway, overexpression of DLX1 was found to increase the protein level of β-catenin, indicating that down-regulation of Wnt/β-catenin was implicated in the miR-4429/DLX1-mediated events.

## Conclusion

**Fig. 7:**
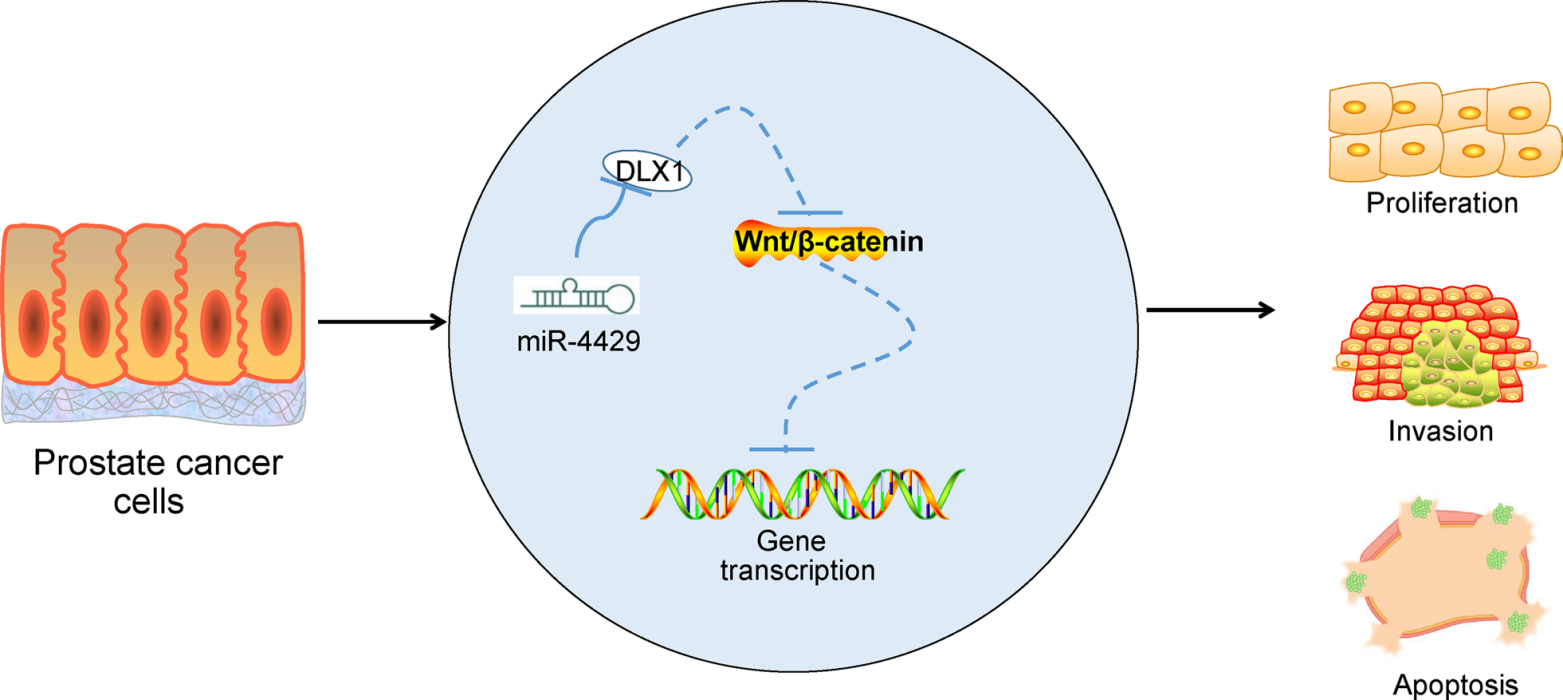
A diagram for the molecular mechanism. miR-4429 inhibits PCa growth through inhibiting the DLX1/Wnt/β-catenin axis

To sum up, our study reports that miR-4429 is lowly expressed in PCa tissues and cells compared to the normal healthy ones. Overexpression of miR-4429 can inhibit PCa growth through regulation of DLX1/Wnt/β-catenin axis (Fig. 7). These finding suggest that low expression of miR-4429 may serve as an indicator for PCa prediction and diagnosis, and miR-4429 may serve as a potential tool for PCa cancer intervention. We also hope that more researches in this field be performed to provide more insights and help the development of novel less-invasive treatments for PCa.

## Supplementary Information

**Additional file 1: Figure S1–S4.** Full-length western blots for Fig. 3f, Fig. 4a, Fig. 4b and Fig. 5a.

## Data Availability

All the data generated or analyzed during this study are included in this published article. Additional data/files would be available from the corresponding author upon reasonable request.

## Abbreviations

ANOVA: Analysis of variance
BSA: Bovine serum albumin
CCK-8: Cell Counting Kit-8
ccRCC: Clear cell renal cell carcinoma
DLX1: Distal‐less 1
FBS: Fetal bovine serum
GAPDH: Glyceraldehyde phosphate dehydrogenase
miRNAs: MicroRNAs
Pca: Prostate cancer
NCs: Negative controls
PBS: Phosphate buffered saline
UTR: Untranslated region
