# Supplementary material for: MicroRNA-4429 suppresses proliferation of prostate cancer cells by targeting distal-less homeobox 1 and inactivating the Wnt/β-catenin pathway
Source: BMC Urol. 2021 Mar 19;21:40. doi: 10.1186/s12894-021-00810-x (PMC7980590; doi:10.1186/s12894-021-00810-x)

Supplemrntary Fig S1

Original western blots for Figure 3F

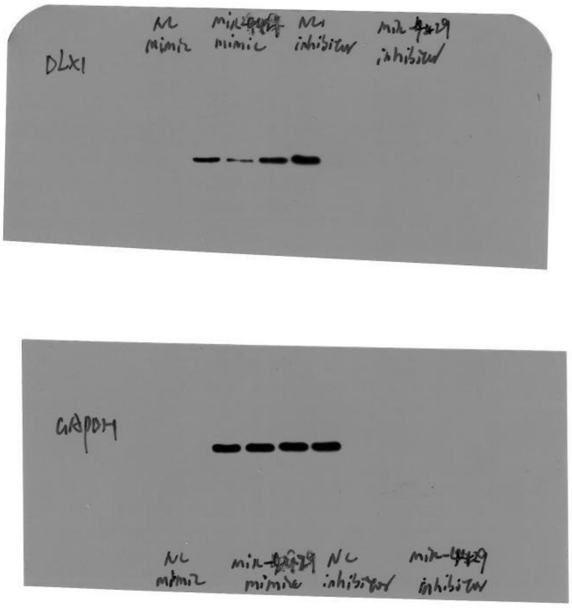

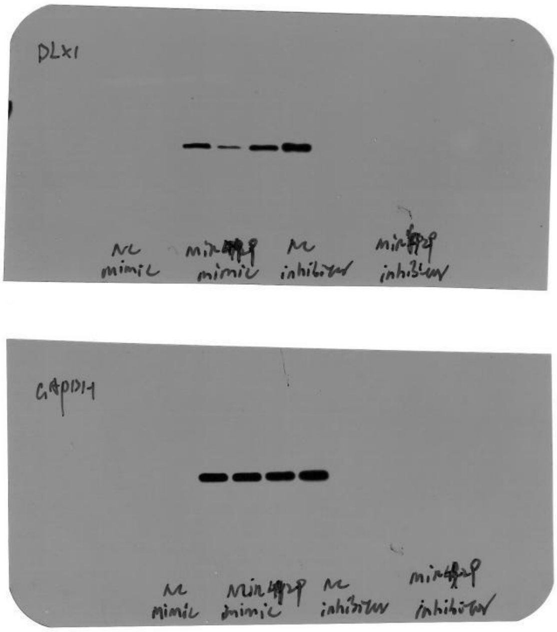

Supplemrntary Fig S2

Original western blots for Figure 4A

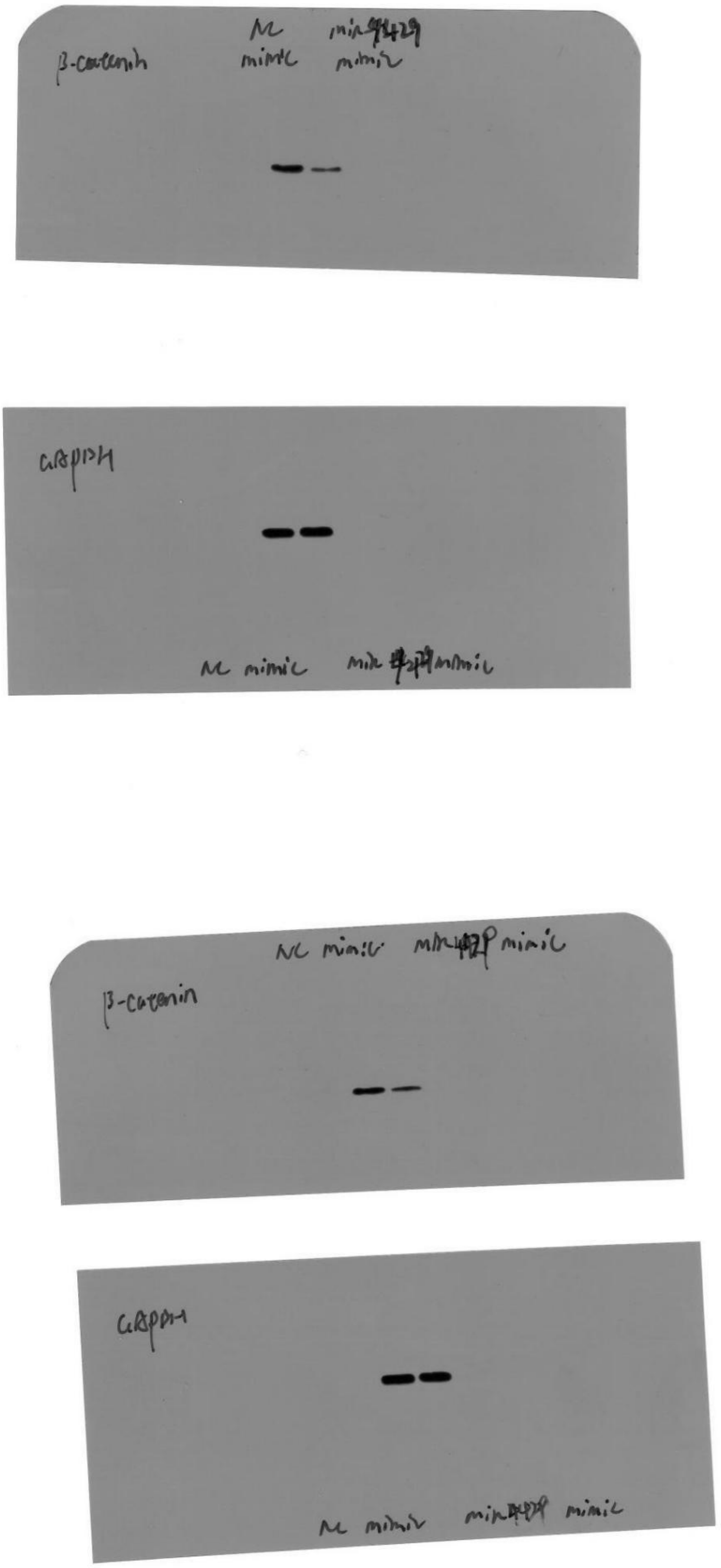

Supplemrntary Fig S3

Original western blots for Figure 4B

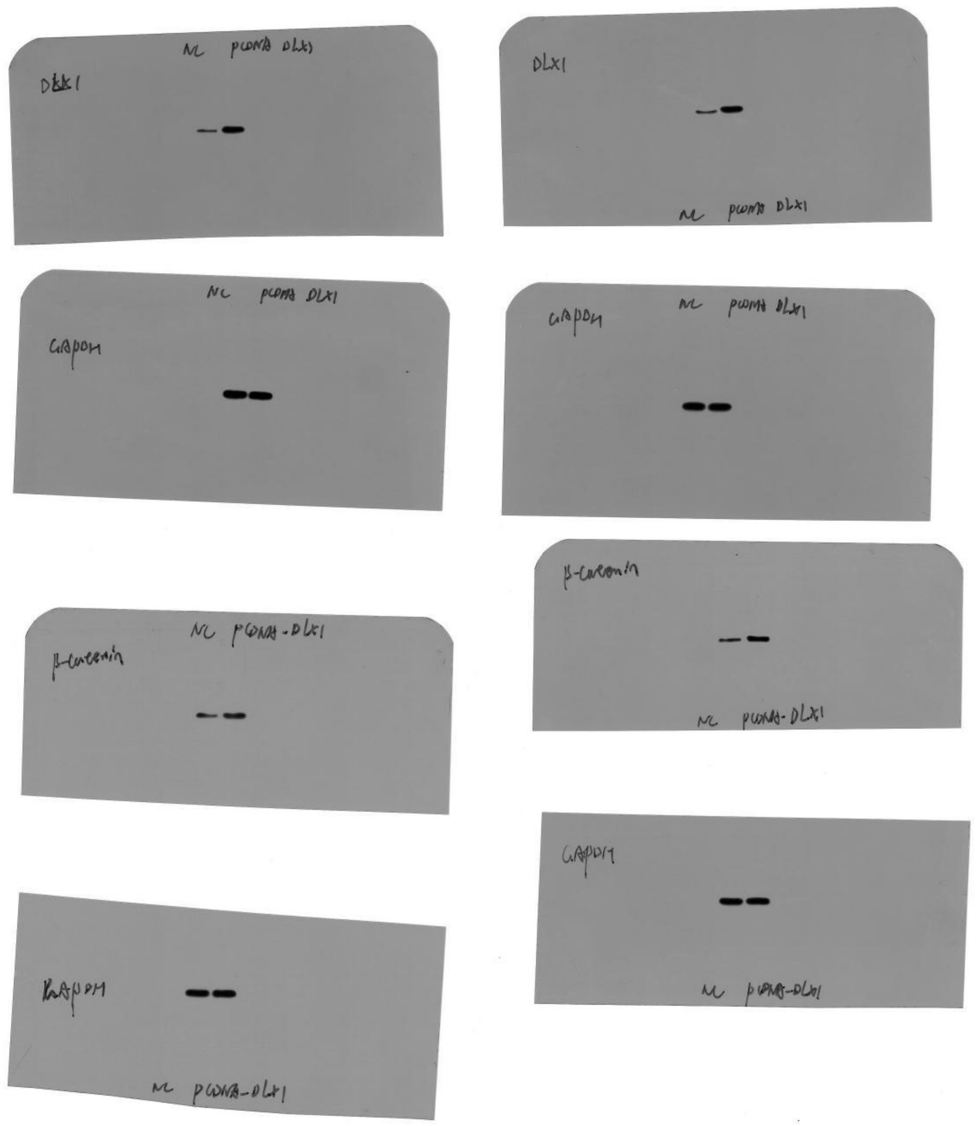

Supplemrntary Fig S4

Original western blots for Figure 5A

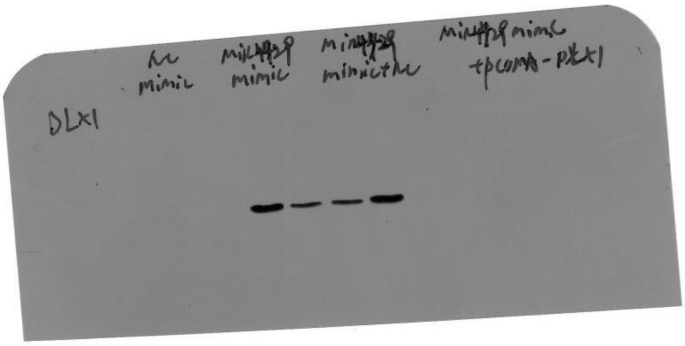

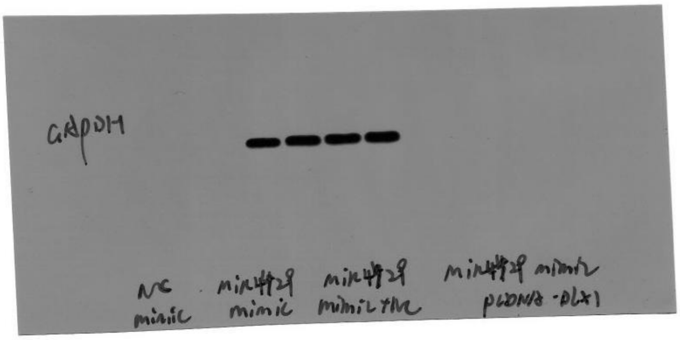

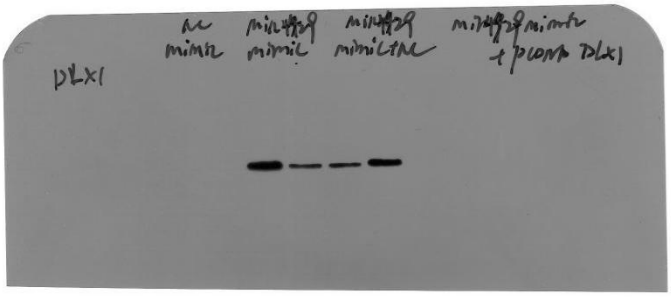

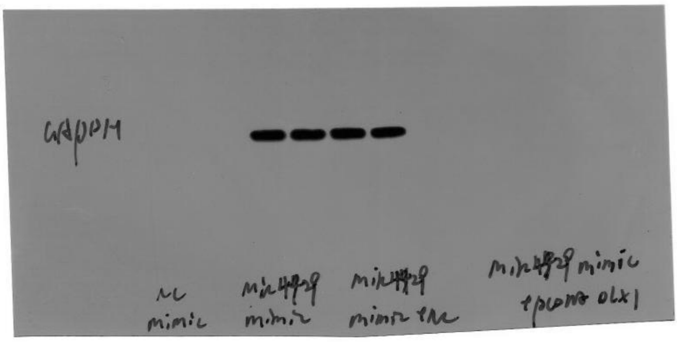

Supplement: Supplementary file 1 — Additional file 1: Figure S1–S4. Full-length western blots for Fig. 3f, Fig. 4a, Fig. 4b and Fig. 5a. [file 12894_2021_810_MOESM1_ESM.pdf]
